# Seasonal variation in morphotype composition of pelagic *Sargassum* influx events is linked to oceanic origin

**DOI:** 10.1038/s41598-023-30969-2

**Published:** 2023-03-07

**Authors:** Kristie S. T. Alleyne, Donald Johnson, Francis Neat, Hazel A. Oxenford, Henri Vallѐs

**Affiliations:** 1 WMU-Sasakawa Global Ocean Institute, World Maritime University (WMU), Malmӧ, Sweden; 2 Center for Fisheries Research & Development, The University of Southern Mississippi-Gulf Coast Research Laboratory, Ocean Springs, MS USA; 3 Centre for Resource Management and Environmental Studies, The University of the West Indies, Cave Hill Campus, Bridgetown, Barbados; 4 Department of Biological and Chemical Sciences, The University of the West Indies, Cave Hill Campus, Bridgetown, Barbados

**Keywords:** Environmental impact, Physical oceanography, Climate-change impacts

## Abstract

The recent proliferation of pelagic *Sargassum* spp. in the Tropical Atlantic causes major ecological and socioeconomic impacts to the wider Caribbean when it washes ashore, with regional fisheries and tourism industries particularly affected. The Caribbean influxes have been tracked to a new bloom region known as the North Equatorial Recirculation Region (NERR) encompassing the area between the South Equatorial Current and the North Equatorial Counter Current and extending from Africa to South America. The vast biomass of *Sargassum* presents serious problems when it washes ashore but also represents significant commercial opportunities, especially with biofuel and fertilizer. The floating *Sargassum* mats are themselves diverse ecosystems that vary both in their biodiversity and biochemical attributes. Two major species (*Sargassum fluitans* and *S. natans*) have been identified as well as several distinguishable morphotypes of each. Oceanic mixing tends to blend the morphotypes together making it difficult to determine if there are regions of the NERR that favour bloom and growth of the distinct types. In this study, we quantify the species and morphotype composition of *Sargassum* strandings in Barbados and test if this is related to separate oceanic origins and routes travelled using a backtracking algorithm based on ocean drifter data. We found significant seasonal variation in the relative abundance of three morphotypes and this could be traced to two distinct easterly sub-origins and/or transport pathways; one area around 15° N that travels directly E–W across the Atlantic, and another area generally south of 10° N that takes a more meandering route coming close the coast of South America. These findings contribute towards our understanding of why the Tropical Atlantic bloom is presently occurring as well as towards addressing valorisation constraints surrounding variation in the supply of the three commonly occurring morphotypes.

## Introduction

Pelagic *Sargassum* spp. (*Sargassum natans* and *S. fluitans*) subsequently referred to simply as ‘*Sargassum*’ forms a floating oceanic ecosystem that provides habitat, shelter and foraging opportunities for a wide diversity of endemic and associated species^1–3^. Historically, this structural habitat was largely confined to the Sargasso Sea and the Gulf of Mexico^4,5^ with very low abundance in the northern Caribbean and Tropical Atlantic^6,7^. This dramatically changed in 2011, when massive quantities of *Sargassum* began to strand and decompose along the coasts throughout the Caribbean, NE South America and West Africa^8–10^ seriously impacting regionally important industries. This proliferation of *Sargassum* in the Tropical Atlantic has continued and increased, and has been linked to a bloom region bounded latitudinally by the South Equatorial Current (SEC) and the North Equatorial Counter Current (NECC) lying between Africa and South America and defined, for simplicity, as the North Equatorial Recirculation Region (NERR)^9,10^. Ocean eutrophication and climate change are likely driving factors^11–13^ although little is known about the effects of ocean circulation patterns on the taxonomic composition of *Sargassum* blooms. In this study, the distribution of *Sargassum* morphotypes within the Tropical Atlantic is determined as a first step in unravelling this complex problem.

The negative impacts of decomposing *Sargassum* have been well documented in nearshore ecosystems^8,14^, fisheries^15–17^, tourism and other coastal businesses^8,18,19^ and human health^20–22^. Clean-up efforts have cost hundreds of millions of dollars to national economies^8,23^. Influx events are now considered a new norm to which countries must adapt^12,24^. There is a rapidly growing interest in utilizing *Sargassum* and turning it into business opportunities^25–28^. However, what constitutes sustainable harvesting and the human and/or environmental health risks of utilizing *Sargassum* are currently not well understood^29^.

**Figure 1:**
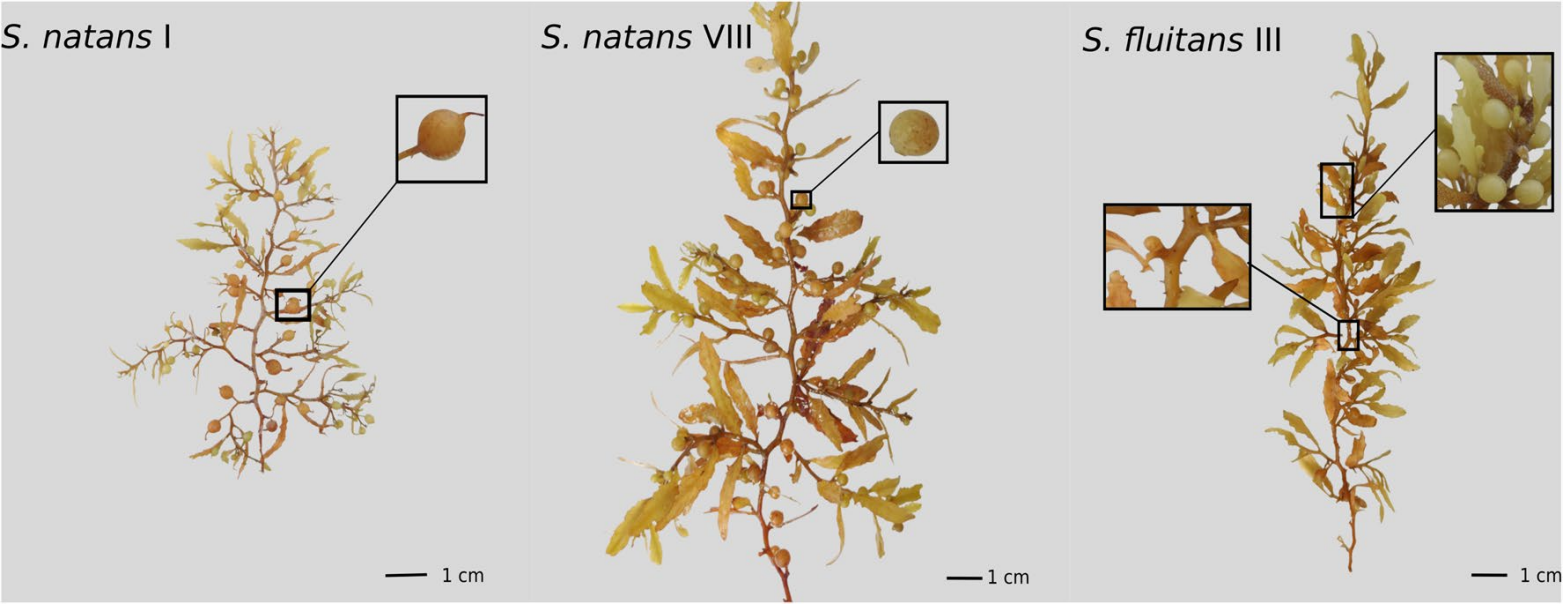
General appearance of the three pelagic *Sargassum* morphotypes collected on Morgan Lewis beach, Barbados. Inset on *S. natans* I shows the presence of spines on the circular air bladders. Inset on *S. natans* VIII shows the absence of spines on the circular air bladders. The top right inset on *S. fluitans* III shows the absence of spines on the elongated air bladders and the bottom left inset shows the presence of spines along the stem. Photograph taken from Ref.^38^.

*Sargassum* mats originating in the Tropical Atlantic are widely recognised as composing of two species (three dominant morphotypes); *Sargassum natans* I, *S. natans* VIII and *S. fluitans* III (Fig. 1). Whilst there remains some controversy over the taxonomy and nomenclature^30–34^, these three morphotypes are genetically distinct^30^ and have distinctive ecological, biological, and chemical traits^3,35^ and even accumulate toxic heavy metals at different rates^36,37^.

There is evidence of substantial interannual and/or intra-annual variation in the morphotype composition of *Sargassum* mats originating from the NERR^29,39–41^. Initial influxes were reported to be dominated by *S. fluitans* III and *S. natans* VIII, whilst more recent observations report a dominance of *S. fluitans* III and *S. natans* I^29,40^. To date, however, the variability in relative abundance of *Sargassum* morphotypes remains poorly understood^29^. Furthermore, given that morphotype composition can influence both the biodiversity associated with *Sargassum*^3^ and chemical composition^35,37^, variations in the relative abundances of the three commonly occurring morphotypes will have implications for sustainable harvesting strategies and for the utilization of *Sargassum* by entrepreneurs.

Recent findings^42^ revealed two potential pathways for *Sargassum* transport into the Caribbean. We hypothesised that the morphotype composition of *Sargassum* influx events varies seasonally and that this is related to its oceanic origin and transport pathway across the Atlantic. We assessed variation in relative abundance of the three morphotypes in *Sargassum* strandings in Barbados during 2021–2022 and analysed their origins with the use of a backtracking algorithm based on ocean drifter data with addition of 0.5% wind^43^. Barbados is uniquely positioned as a study site, being near the 15° N latitude ‘separation’ line between North Atlantic gyre water and tropical water entering the Caribbean^44^ and the most easterly of the Caribbean islands, thus among the first to receive *Sargassum* influxes from the NERR. Therefore, it serves as an ideal site to test our hypothesis regarding seasonal variation and oceanic origins of *Sargassum* influxes.

## Results

### Changes in morphotype compositions

**Figure 2:**
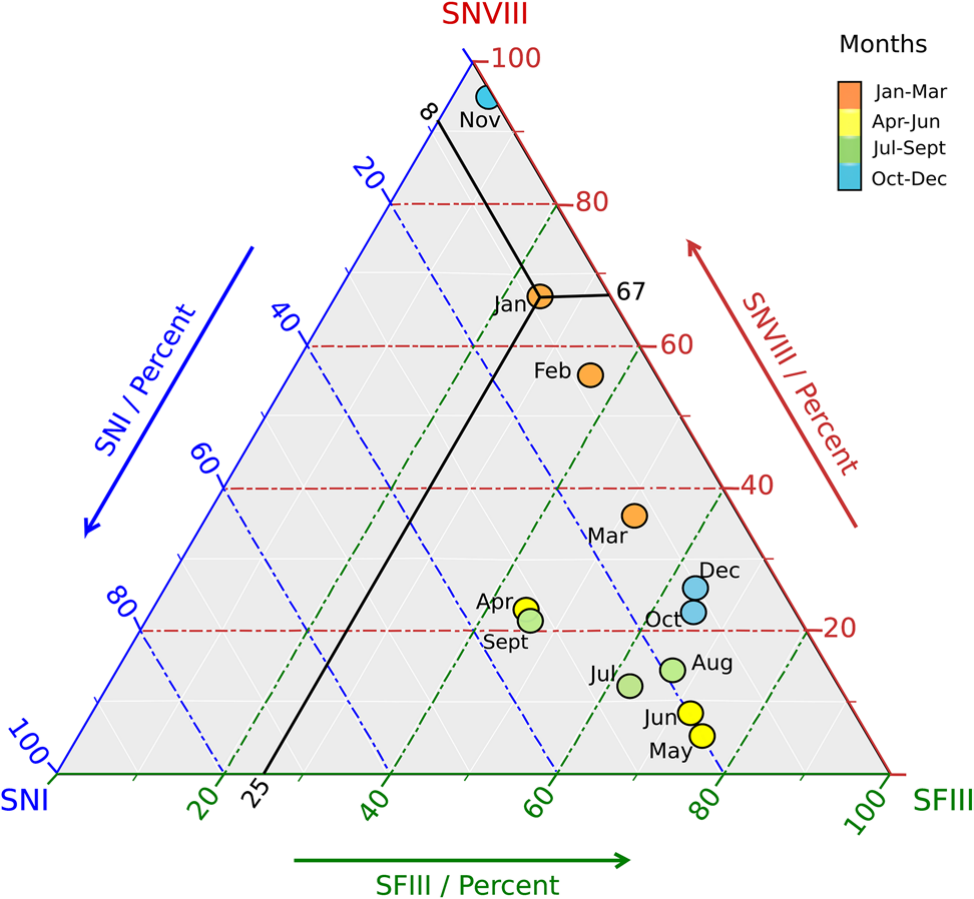
Ternary plot illustrating monthly variation in morphotype composition of *Sargassum* stranding in Barbados over a 12-month period. *Sargassum natans* I (SNI), *S. natans* VIII (SNVIII) and *S. fluitans* III (SFIII). Black lines indicate the average percent contribution of each morphotype in the January samples (67% SNVIII, 25% SFIII, 8% SNI). Quarterly periods are represented by orange (Jan–Mar), yellow (April–Jun), green (Jul–Sept) and blue (Oct–Dec) dots.

Monthly analyses of the relative abundance of *S. natans* I, *S. natans* VIII and *S. fluitans* III revealed temporal differences in the predominant morphotype (Fig. 2, Fig. S1).

While *S. fluitans* III was the predominant morphotype in 80% of the samples, there were notable exceptions (Fig. 2). For example, in November, January, and February samples were dominated by *S. natans* VIII, which was generally the least abundant of the three morphotypes.

### Linking morphotype compositions to sub-origins

**Figure 3:**
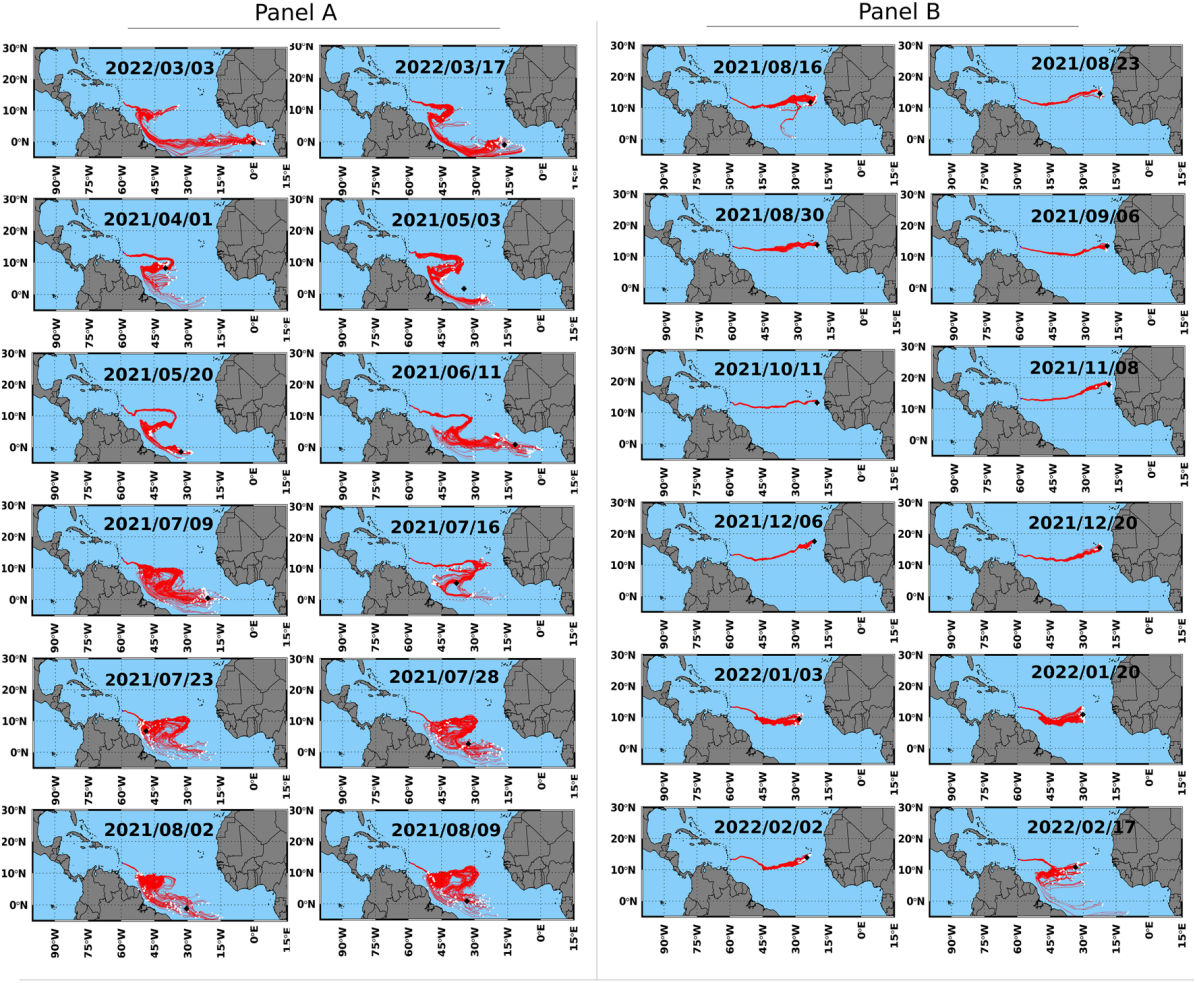
Backtracks of *Sargassum* (mixed-morphotype samples) collected from Morgan Lewis beach in Barbados over the period of 1 year (2021–2022). Panel (**a**) shows sample backtracks from early March to early August and panel (**b**) shows backtracks from late August through February. All backtracks were run over a 365-day period using 100 particles. White dots indicate the origin of each backtrack particle (i.e., the backtrack location 365 days before arriving to Barbados) and the black square represents the origin of highest probability (mean of all backtrack particles). Dates are presented in year/month/day format. Maps were generated using IDL 8.8.1 (https://www.l3harrisgeospatial.com/Software-Technology/IDL).

The extent to which the observed variation in the relative abundance of *Sargassum* morphotypes reflects distinct origins was investigated via sample backtracking for 365 days from the date of stranding using 100 particles to represent each sample. This revealed two distinct sub-origins/transport pathways (Fig. 3). *Sargassum* stranding in Barbados between March and early August apparently originated close to the equator and travelled along northeast Brazil before arriving in Barbados (Fig. 3a). In contrast, *Sargassum* arriving between late August and February originated further north and travelled a relatively direct route to Barbados (Fig. 3b).

**Figure 4:**
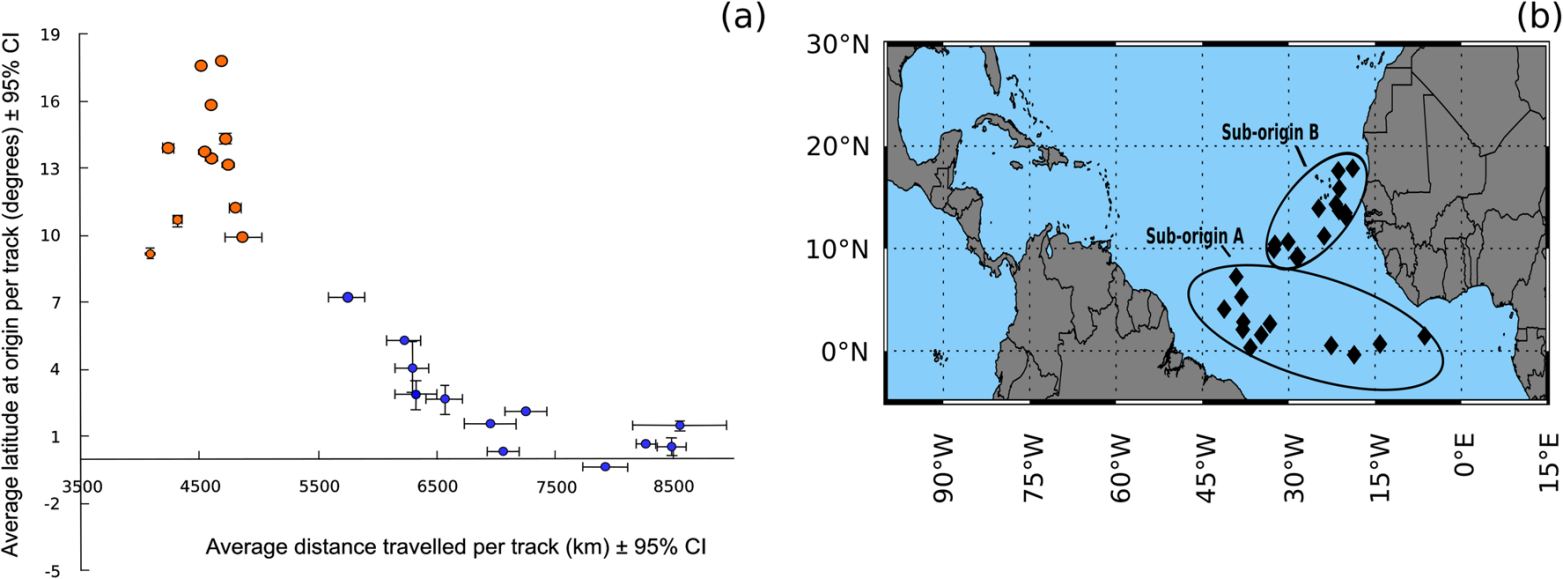
*Sargassum* sub-origins and transport pathway metrics within the Tropical Atlantic as determined from backtracking monthly *Sargassum* samples stranded in Barbados. Part (**a**) shows the average (± 95% CI) distance travelled per track and the average (± 95% CI) latitude of each origin for a given date. Blue dots indicate samples from sub-origin A and orange dots indicate samples from sub-origin B. Part (**b**) shows the two distinct sub-origins identified. Black square represents the average latitude at the origin of each track for a given date. Map was generated using IDL 8.8.1 (https://www.l3harrisgeospatial.com/Software-Technology/IDL). All backtracks started from the Morgan Lewis beach, located on the east coast of Barbados and were run over a 365-day period using 100 particles. All averages represent mean value of the 100 particles.

The two identified transport pathways differed in the average distance travelled by the particles and average latitude at the origin of each particle backtrack (Fig. 4a). Simulations were attributed to sub-origin/transport pathway A or B based on the route travelled, distance travelled and the approximate location of the origins. Backtracks that originated around the equator (0–7° N; Fig. 4b) and followed a convoluted trajectory along the NE coast of Brazil to the Caribbean (Fig. 3a) are referred to as sub-origin/transport pathway A. These backtracks showed both high average distances travelled (5740 to 8550 km over the 365 days) as well as high variability in the distance travelled among replicate backtracks for any given date (indicated by the relatively large 95% confidence intervals in Fig. 4a). In contrast, backtracks referred to as sub-origin/transport pathway B originated relatively far north (9–18° N; Fig. 4b) and travelled a more direct westerly route covering a much shorter distance over the 365 days (4080–4870 km) than *Sargassum* from sub-origin/transport pathway A (Fig. 3a,b) and showed greater consistency in the distance travelled among the replicate backtrack particles for any given date (Fig. 4a). This partitioning of the 25 simulations into two “homogenous” groups of sub-origins was supported with the use of K-means partitioning (Fig. S2).

**Figure 5:**
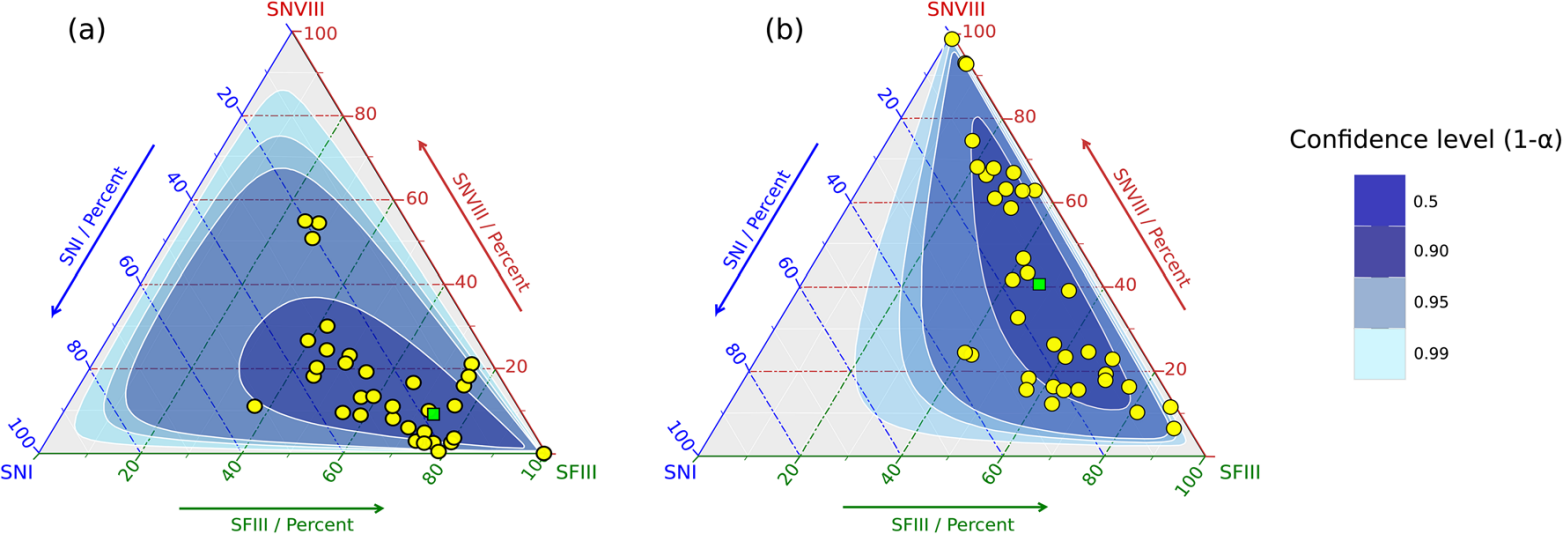
Ternary plots showing morphotype composition of *Sargassum* samples stranding in Barbados over a 12-month period. Part (**a**) shows *Sargassum* samples from sub-origin/transport pathway A (March–early August). Part (**b**) shows *Sargassum* samples from sub-origin/transport pathway B (late August–February). *Sargassum natans* I (SNI), *S. natans* VIII (SNVIII) and *S. fluitans* III (SFIII). Samples are represented by yellow dots and the mean value is represented by a green square.

A Permutational Multivariate Analysis of Variance (PERMANOVA) indicated that the relative abundance of *S. natans* I, *S. natans* VIII and *S. fluitans* III morphotypes differed significantly between the two sub-origins/transport pathways (p = 0.0131) (Table S1). Sub-origin/transport pathway A (March-early August) showed a predominance of *S. fluitans* III (Fig. 5a). In contrast, sub-origin/transport pathway B (late August–February) showed higher levels of *S. natans* VIII, with reduced quantities of *S. fluitans* III (Fig. 5b).

### Oceanographic parameters and sub-origins

**Figure 6:**
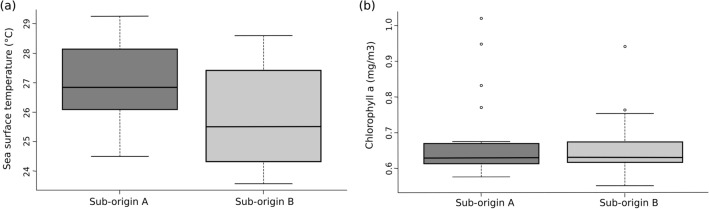
Boxplots of sea surface temperature (**a**) and chlorophyll a concentration (**b**) for sub-origin/transport pathway A and B (n = 48 in all groups).

We investigated whether environmental conditions, i.e., Sea Surface Temperature (SST) and nutrient load (inferred from Chlorophyll a concentration) derived from satellite imagery, differed between the two sub-origins at the initial locations (‘origins’ (Fig. 4b)) and mid-way (6 months) along the average backtrack trajectory of *Sargassum* (for a given date). A PERMANOVA indicated that environmental conditions differed significantly between the two sub-origin/transport pathways (p = 0.0039) but not between time periods (origin vs mid-way) (p = 0.3179) (Table S1). Pooling the data across both time periods for each sub-origin supported that environmental differences between the two sub-origins/transport pathways were driven by higher SST at the sub-origin/pathway A (Fig. 6).

## Discussion

Since the onset of *Sargassum* influx events in 2011, there has been notable annual variability in the relative abundance of *S. natans* I, *S. natans* VIII, and *S. fluitans* III, with broadscale spatial and temporal differences observed across the Caribbean^29,39–41^; however, to date there has been little understanding of what drives this. This study supports for the first time that the morphotype composition of *Sargassum* influxes over the course of a full year varies significantly and appears to be linked to their origin/transport pathway. By using a backtracking algorithm based on ocean drifter data with 0.5% wind, *Sargassum* from Barbados was traced to two distinct sub-origins/transport pathways within the Tropical Atlantic. Identified pathways align with the findings of Ref.^42^. In our assessment, *Sargassum* mats arriving in Barbados between March to early August are likely to take the more southerly transport pathway, linked to the Gulf of Guinea, (arriving from sub-origin A) that passes along the coast of South America. Satellite imagery in Refs.^7,45^ suggest that *Sargassum* enters this pathway from south of the equator, which is south of the NERR. Alternatively, *Sargassum* arriving between late-August and February are likely to take the more northerly transport pathway (arriving from sub-origin B). This matches the seasonal distribution of *Sargassum* in the NERR as detected by satellites^13^. *Sargassum* arriving from sub-origin/transport pathway A were *S. fluitans* III-dominated while those arriving from sub-origin/transport pathway B had significantly higher amounts of *S. natans* VIII.

Interestingly, our monthly observations of relative abundance of the three *Sargassum* morphotypes aligns with morphotype compositions reported in the literature^39–41^. Starting with the November 2014 to May 2015 period^41^, *S. natans* VIII was the predominant form observed across the Caribbean. This was especially interesting since no earlier studies had ever reported *S. natans* VIII-dominated mats throughout the region. Subsequent studies^39^ in the Mexican Caribbean, also reported large volumes of *S. natans* VIII during the peak arrival of *Sargassum* in August 2015. In contrast, recent investigations into the morphotype composition arriving in Jamaica during July and August^40^ showed a clear dominance of *S. fluitans* III. Based on the findings of our study, a possible explanation for the large-scale variations observed by Refs.^39–41^ can be linked to the identified sub-origins/transport pathways. It is plausible that 2015 reports of *Sargassum*^39,41^ recorded high amounts of *S. natans* VIII because the majority of their data collection took place during the late August-February (sub-origin/transport pathway B) period, which was associated with significantly higher abundances of *S. natans* VIII in our analyses. On the other hand, the 2020 summer report^40^ of *Sargassum* influx events reported dominance of *S. fluitans* III; according to the findings of the current study *Sargassum* mats arriving during this time would have likely arrived from sub-origin/transport pathway A, which shows a significantly higher abundance of *S. fluitans* III.

While the presence of a southern sub-origin/transport pathway A and a northern sub-origin/transport pathway B provides a plausible explanation for the observed spatial and temporal variations in the relative abundances of *S. natans* I, *S. natans* VIII and *S. fluitans* III, the question remains: why are these sub-origins/transport pathways favouring different morphotypes? In Florida, the two *Sargassum* species are reported to have different maximum growth rates under optimal conditions, with *S. fluitans* apparently capable of growing considerably faster than *S. natans*^46^. The same study also indicated a difference in thermal tolerances between the two species, stating that *S. natans* has a broader optimal temperature range (18–30 °C) than *S. fluitans*. Furthermore, recent studies have also indicated similar differences in growth rates between species, as well as differences among morphotypes from the Tropical Atlantic^47^. Studies by Lapointe and colleagues comparing growth rates have also indicated that both pelagic species grow significantly faster with nutrient enrichment^5,48,49^. Therefore, given the two distinct sub-origins/transport pathways, oceanographic conditions between the two areas may have been the proximal cause for the observed morphotype compositions. Our analysis suggests that differing SST within sub-origins/transport pathways influenced the morphotype composition of *Sargassum* arriving in Barbados. *S. fluitans* III-dominated mats arriving from the southerly transport pathway A, close to the equator, experienced higher SST when compared to the northerly transport pathway B. Recent studies^12^ indicate that *Sargassum* blooms are enhanced by nitrogen-rich neritic waters and that nutrient variability is a key driver of *Sargassum* variability^13^. In this study we used chlorophyll a concentration as a direct proxy for nutrient concentration. However, we found high variability in this proxy within each of the two sub-origins/transport pathways, which precluded a clear understanding of its possible role on morphotype composition. Moreover, it is possible that chlorophyll concentration per se might not be a good indicator of nutrient availability for *Sargassum*. The Equatorial Atlantic has a heterogenous surface environment with contributing nutrient-rich water masses from the Amazon River, the Congo River and equatorial and coastal upwelling^50,51^. Thus, mats originating close to the equator (sub-origin/transport pathway A) are likely to experience nutrient-rich environments in addition to higher SST than *Sargassum* from the more northerly sub-origin/transport pathway B, allowing *Sargassum* to flourish and perhaps amplifying physiological differences among morphotypes that result in differential growth and mortality and thus in different morphotype composition. However, discussions surrounding optimal growth conditions for the three morphotypes remain largely speculative and the extent to which the rate of nutrient uptake varies among morphotypes is not well understood. Interestingly, *S. natans* VIII and *S. fluitans* III collected from around 10° N showed different levels (albeit not significant) of %N enrichment; with *S. natans* VIII having higher %N on average than the other two morphotypes^12^. If *S. natans* VIII is indeed capable of absorbing nutrients faster than its counterparts above 10° N, valorisation of this morphotype may be affected. Low phosphate environments increase the uptake of arsenic^37^, therefore, mats traversing low phosphate environments at and above 10° N may result in *S. natans* VIII absorbing higher levels of arsenic compared to *S. natans* I and *S. fluitans* III, ultimately reducing the applications for which *S. natans* VIII can be used. Intriguingly, biomass composition of *Sargassum* arriving in Jamaica during February 2019 had lower quantities of metals in *S. natans* VIII than *S. natans* I and *S. fluitans* III; but when looking at arsenic specifically, *S. natans* I and *S. natans* VIII on average contained higher levels of arsenic when compared to *S. fluitans* III^35^. Studies on growth and mortality of the three morphotypes in various conditions are required to understand their optimal conditions, the effects of source and dispersal routes on morphotype composition, biomass composition and the potential consequences for valorisation.

The observed seasonality *in S. fluitans* III and *S. natans* VIII may also influence the biodiversity associated with *Sargassum* mats at different times of the year. The greater structural complexity of *S. fluitans* III supports more organisms when compared to the less foliated *S. natans* VIII^3^. This means that *S. fluitans* III-dominated mats arriving from sub-origin/transport pathway A during March to early August may have greater biodiversity than mats arriving from sub-origin/transport pathway B (late August to February). With more innovative strides being made towards *Sargassum* valorisation^26^, in-water harvesting may be the solution to providing large quantities of fresh *Sargassum* for a variety of uses. However, in-water harvesting may pose a threat to associated biodiversity especially during months where *S. fluitans* III is the dominant morphotype. To better understand the implication(s) of changing morphotype composition on *Sargassum* associated biodiversity and valorisation efforts, further research into the seasonality of *Sargassum* morphotypes is required. Studies should ideally be conducted across the Caribbean to provide a comprehensive understanding of the situation and aid in the region’s continued adaptation to *Sargassum* influx events.

There are limitations to our study. As such generalizations within or across years or countries should not be made with only one year of data. We cannot say if the results of 2021–2022 reflect a typical year or if the findings were unique to Barbados given that it is one of the first islands to receive *Sargassum* from the NERR. As *Sargassum* travels from eastern to western countries across the Caribbean and experiences different environments, differential growth and mortality will undoubtedly play a role in *Sargassum* quantities and morphotype composition. A further potential limitation of this study arises from the simple classification of simulations into sub-origin/transport pathway A and B. This simple classification into two pathways points to resolvability of the complex problem of blooms; why now, why here, what are the dominant parameters that influence growth and mortality? Nevertheless, the findings of this study are relevant to the developing *Sargassum* industry by providing insights into the potential causes of variation in morphotype composition arriving in the Caribbean; and by extension provide a baseline for further studies on the predictability of seasonal patterns. The identification of the two sub-origins/transport pathways has implications for advancing the region’s understanding of the factors responsible for the continued proliferation and extensive interannual variability of *Sargassum* in the Tropical Atlantic since the initial bloom in 2011.

## Methods

### Sample collection and sorting

For each date of sampling effort, three clumps of newly beached, wet, “fresh gold” *Sargassum* were collected from Morgan Lewis beach, Barbados (13° 16′ 4.86′′ N–59° 33′ 48.41′′ W) (Fig. S3). Clumps (~ 0.27 kg each) were collected using both hands at approximately 10 m intervals along the shoreline. Using gross morphological features (following^52^), each of the three clumps was carefully separated into its component morphotypes, *Sargassum natans* I, *S. natans* VIII and *S. fluitans* III (Fig. 1). The displacement volume of each morphotype from each clump was then obtained using a measuring cylinder filled with a known volume of seawater. Sampling occurred opportunistically from February 2021 to March 2022, with at least one sample being collected each month. Sampling efforts resulted in 24 collection days over a 1-year period.

### Backtracking of pelagic *Sargassum*

Satellite tracked mix-layer drifters (drogue element at 15 m) from the Global Drifter Program (GDP)^53^ have been deployed around the globe since ~ 1979. These drifters provide reliable tracking of water particles at drogue depth and are equipped with batteries that can last in excess of 450 days; however, loss of drogue is common. Using a Surface Velocity Program (SVP), data are provided on position, temperature and drogue on/off^54^. Current vector components are calculated at 6-h intervals from sequential positions and can be retrieved from https://www.aoml.noaa.gov/phod/gdp/. This study used a compiled file of the GDP data set consisting of: year, day, hour, longitude, latitude, east-current, west-current and drogue-on/drogue-off flag from 1979 to 2020. For tracking, the data were interpolated to a 1/12th degree resolution grid at 365 year-day intervals. The exact steps taken to achieve this dataset can be found in Ref.^43^.

To determine potential origins of the sampled *Sargassum*, each of the 24 collection days were backtracked using IDL 8.8.1 programming software (https://www.l3harris.com/all-capabilities/idl). Using a simple backtracking algorithm based on the drifter data set and 0.5% windage, *Sargassum* was tracked back 365 days from collection points on Morgan Lewis beach to determine the origin of each sample within the Tropical Atlantic. The selected 365-day time for backtracking was based on an experiment that assessed the dispersion of *Sargassum* from the NERR (Fig. S4). Our results showed that approximately 90% of the *Sargassum* population within the NERR is dispersed within 365 days. Recent studies^44^ also found that *Sargassum* present in the NERR has a high probability of entering the Caribbean within a year’s time.

Backtracks were simulated using 100 particles launched simultaneously from the collection location at Morgan Lewis beach, applying sub grid-scale turbulent motions (Lagrangian Stochastic Model^55^) to each particle’s current component:

1 $${\text{u}}^{\prime } = {\text{ u }} + \, 0.{1 } \times {\text{ current speed }} \times {\text{ P(1)}}$$

where u′ is an adjusted current component and P(1) is a normal (Gaussian) random distribution with a mean of zero and a standard deviation of one. This simple turbulence addition to each of the east–west (u) and north–south (v) current components acknowledges that the gridded current database is smoother than reality. End points of each particle’s back-trajectory were obtained by center-of-mass calculations of the 100 ending locations.

The tracking methodology used in this study was developed by Ref.^43^ and is currently used to obtain 3-month *Sargassum* forecasts for the Lesser Antilles, published in the Sargassum Sub-Regional Outlook Bulletin (https://www.cavehill.uwi.edu/cermes/projects/sargassum/outlook-bulletin.aspx). Forecasts from the Outlook bulletin are well suited for monitoring *Sargassum* within the Lesser Antilles and are in close agreement with observed influxes^56^.

### Data analysis

Monthly changes in relative abundance of *Sargassum* morphotypes (volume of morphotype/total volume of *Sargassum* sample) were calculated by averaging compositional sample data first by date (if multiple *Sargassum* samples were collected the same date) and then by month (if multiple dates were sampled within the same month). Differences in relative abundance of *Sargassum* morphotypes between the two sub-origins were tested using a PERMANOVA with the sample compositional data (transformed into a bivariate matrix following^57^ to address non-independence of the three percent estimates) as response matrix and sub-origin as independent factor, while implementing a constrained nested permutation scheme with sample data nested within dates and dates serving as independent statistical replicates for each sub-origin. This test was conducted using the adonis function of the “vegan” package in R^58,59^. Data were also checked for homogeneity of dispersion using the betadisper function of the same package (Table S1). Given that *Sargassum* compositional samples involved three morphotypes, we used ternary plots to display these data; these plots were produced using the “ggplot2”, “ggtern”, “ggpubr”, and “lattice” packages in R^59–63^.

Environmental data were retrieved using Giovanni (https://giovanni.gsfc.nasa.gov/giovanni/) time-series area-average oceanic data. The data sources were Sea Surface Temperature at 4 microns (Night) 8-daily 4 km (MODIS-Aqua) and Chlorophyll a concentration 8-daily 4 km (MODIS-Aqua MODISA_L3m_CHL_8d_4km). These data were retrieved for each collection date for (1) the location and time of origin (i.e., 365 backtracked days; 1 year) and the location and time of the mid-point (183 backtracked days; 6 months). For example, when assessing the initial SST conditions at time of origin for a sample collected on the 10th of January 2022 in Barbados, we used the 8-day SST average data from the closest available time intervals the year before (i.e., January 2021) at the estimated point of origin. To do this, a box of approximately 93,500 sq km was drawn centred around the point of origin. Then, the SST 8-day average for the entire box area was downloaded. To test for differences in SST and chlorophyll a between sub-origins a PERMANOVA test using SST and chlorophyll as response bivariate matrix data and time period (initial vs mid-way point) and sub-origin location (A vs B) (and their interaction) as independent factors. This test was also conducted using the adonis function of the “vegan” package in R^58,59^. Chlorophyll a data were square-root transformed to minimize the effect of extreme values. The significance level of 0.05 was used for all analyses.

## Supplementary Information

Supplementary Information 1.

Supplementary Information 2.

Supplementary Information 3.

Supplementary Information 4.

Supplementary Information 5.

## Data Availability

All data generated or analysed during this study are included in this published article [and its Supplementary Information files].
