# Supplementary material for: Seasonal variation in morphotype composition of pelagic Sargassum influx events is linked to oceanic origin
Source: Sci Rep. 2023 Mar 7;13:3753. doi: 10.1038/s41598-023-30969-2 (PMC9992440; doi:10.1038/s41598-023-30969-2)
Supplement: Supplementary file 4 — Supplementary Information 4. [file 41598_2023_30969_MOESM4_ESM.docx]

**Supplementary Information**

Seasonal variation in morphotype composition of pelagic *Sargassum* influx events is linked to oceanic origin.

Kristie S. T. Alleyne^1*^, Donald Johnson^2^, Francis Neat^1^, Hazel A. Oxenford^3^ and Henri Vallѐs^4^

^1^ WMU-Sasakawa Global Ocean Institute, World Maritime University (WMU), Malmӧ, Sweden; w2005361@wmu.se (K.A*); fn@wmu.se (F.N)

^2^Center for Fisheries Research & Development, The University of Southern Mississippi-Gulf Coast Research Laboratory, Ocean Springs, MS, USA; donald.r.johnson@usm.edu

^3^Centre for Resource Management and Environmental Studies, The University of the West Indies, Cave Hill Campus, Bridgetown, Barbados; hazel.oxenford@cavehill.uwi.edu

^4^Department of Biological and Chemical Sciences, The University of the West Indies, Cave Hill Campus, Bridgetown, Barbados; henri.valles@cavehill.uwi.edu

**The Supplementary Information includes:**

Supplementary Figure 1

Supplementary Figure 2

Supplementary Figure 3

Supplementary Figure 4

Supplementary Table 1

**Supplementary Figure 1.** Barchart of monthly relative abundance composition of three commonly occurring *Sargassum* morphotypes (*Sargassum natans* I, *S. natans* VIII, *S. fluitans* III) arriving at Morgan Lewis beach in Barbados, over a one-year period**.**

K-means partitioning was used to identify the best number of “homogenous” groups to allocate the 25 simulated origins. This k-means partitioning was based on three metrics: (1) average simulated distance travelled from an origin to Barbados, (2) average simulated latitude position mid-way through the trajectory (day 186) for an origin, and (3) average simulated latitude position at initial starting point (day 385) for an origin. All three variables were standardized prior to the k-means partitioning to give them equal weight. Figure S2 below show that a partitioning of two “homogenous” groups of origins of roughly similar size provides the best compromise, as evidenced by the highest Calinski criterion value.

**
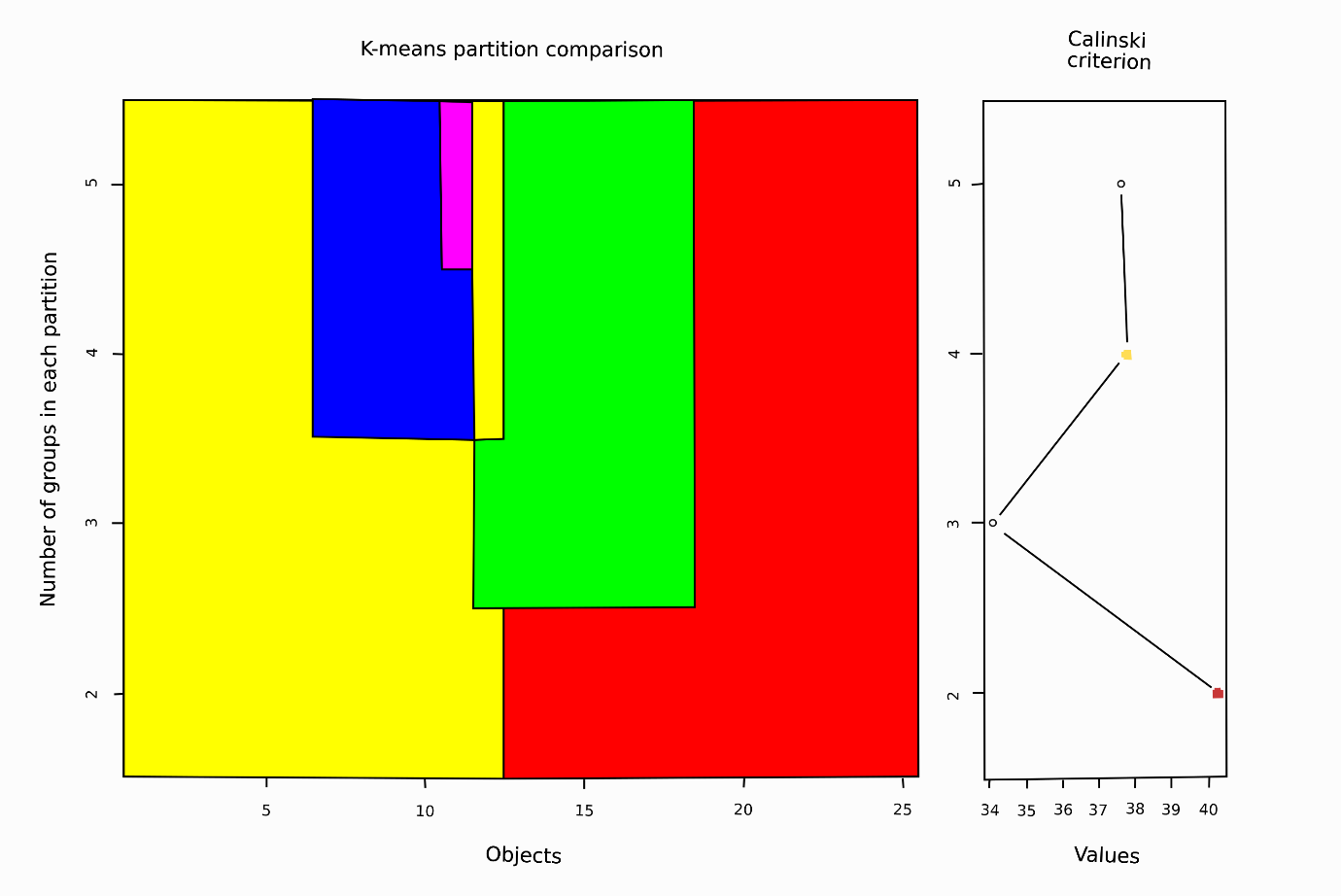
**

**Supplementary Figure 2.** K-means partitioning of the 25 simulated pathways presented in Figure 3 of the main text.


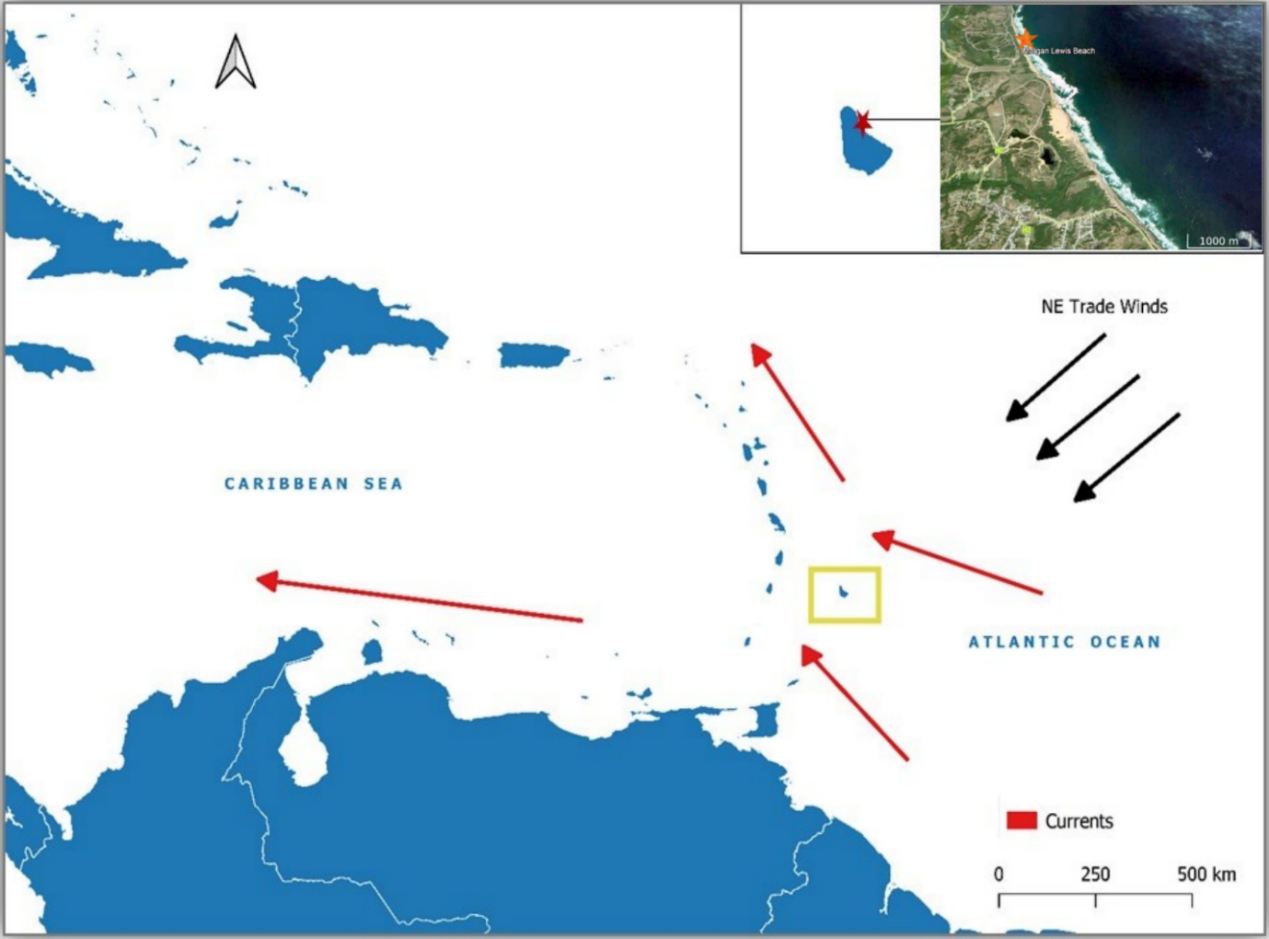


**Supplementary Figure 3.** Map depicting Barbados (yellow box) within the Caribbean region, the region’s prevailing ocean currents (red arrows) and Northeast Trade Winds (black arrows). Sampling location (Morgan Lewis beach) for the collection of stranded sargassum is shown in the inset map. Map was generated using QGIS Desktop 3.16.10 (https://www.qgis.org/en/site/about/index.html) and Natural Earth Data (<https://www.naturalearthdata.com/downloads/10m-cultural-vectors/10m-admin-0-countries/>) was used for the country shapefile. The inset map was generated using Goggle Earth.

In a simple experiment using our drifter current field and a homogeneous distribution of sargassum in the NERR, we found that dispersion dominated consolidation and retention. At the end of 1 year only 11% remained and at the end of 2 years just 1.3% (Supplementary Figure 4). There did not appear to be a significant seasonal signal to this pattern. This rapid dispersal means that ~ 90 % of the sargassum population must be replaced every year, hence 365 days is a reasonable scale to backtrack to ‘origin’.


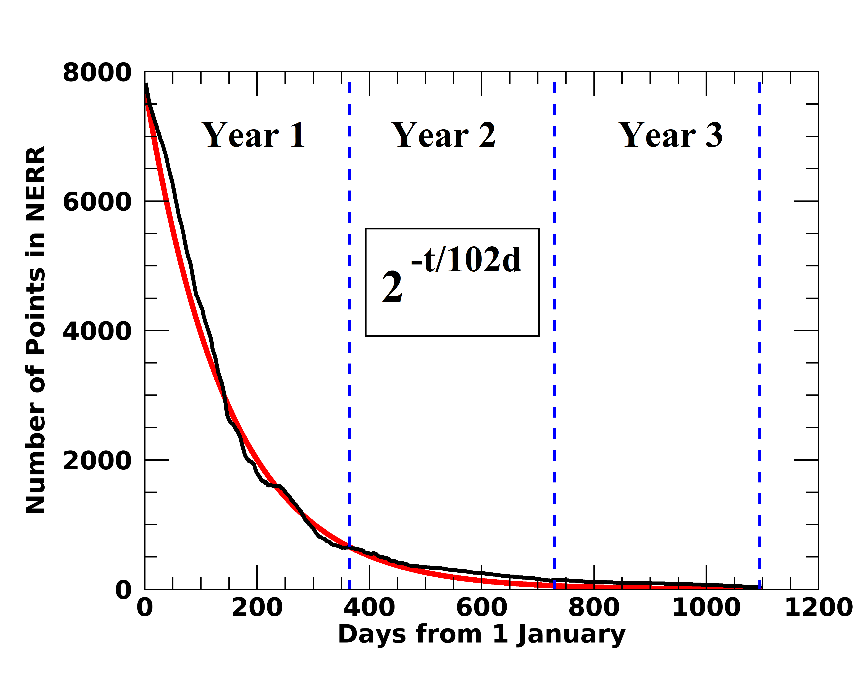


**Supplementary Figure 4.** Results of experiment indicating the dominance of sargassum dispersion from the tropical Atlantic. The red line is best fit of a ‘half-life’ decay (dispersion) rate at 102 days for 0.5% windage (used in our model) – leaving 8.4% of the sargassum population after 365 days. The black line is best fit of a ‘half-life’ decay rate at 114 days for 0.25% windage – leaving 10.9% after 365 days.

**Supplementary Table 1.** Multivariate analyses of morphotype composition, SST and Chlorophyll a between sub-origin A and B. Factors included were ‘location’ (sub-origin A vs. sub-origin B), ‘time period’ (initial and mid-way) and all interaction effects. Significant (<0.05) P values are bold.

| **Test**/Factor(s) | **Description** | **df** | **SS** | **F** | **R2** | **P** | **No. of Perm.** |  |
| --- | --- | --- | --- | --- | --- | --- | --- | --- |
| **Betadisper** | Homogeneity of dispersion between sub-origin A and sub-origin B samples for morphotype composition | 1 | 0.4483 | 1.6639 | - | 0.378 | 999 |  |
|  |  |  |  |  |  |  |  |  |
|  |  |  |  |  |  |  |  |  |
| **Betadisper** | Differences in dispersion between sub-origin A and sub-origin B samples for SST and Chlorophyll a | 1 | 0.082 | 0.1151 | - | 0.756 | 999 |  |
|  |  |  |  |  |  |  |  |  |
|  |  |  |  |  |  |  |  |  |
|  |  |  |  |  |  |  |  |  |
| **PERMANOVA**  Location | Differences in morphotype composition between sub-origin A and sub-origin B. | 1 | 66.09 | 13.947 | 0.16614 | **0.0131** | 9999 |  |
|  |  |  |  |  |  |  |  |  |
|  |  |  |  |  |  |  |  |  |
| **PERMANOVA**    Sub-origin (i.e., A and B)  Time period (i.e., initial and mid-way)    Sub-origin: Time period | Differences in SST and Chlorophyll a at origins and mid-points | 1  1  1 | 7.837  2.601  0.681 | 4.1604  1.381  0.3614 | 0.08337  0.02767  0.00724 | **0.0039**  0.3179  0.7457 | 9999 |  |
